# On Bleeding in Severe Injuries

**Published:** 1817-02

**Authors:** R. N. Starr

**Affiliations:** Member of the Royal College of Surgeons, London.


*For the London Medical and Physical Journal.*

### *On Bleeding in Severe Injuries;* by R. N. STARR, Member of the Royal College of Surgeons, London.

THE statement of the two following cases may, perhaps, appear unnecessary, after what has already been said on the treatment of injuries in general. Yet frequent observation of the success which attends the free use of the lancet in extensive accidents, has induced me to forward the inclosed for the use of the London Medical and Physical Journal.

CASE I.—Jas. Thorp, ætat. 12, on the afternoon of the 10th of Sept. last, received a severe wound on the anterior part of the thorax on the left side, between the third and fourth ribs, by the passing of an iron hook, which was attached to the shaft of a cart. Upon examining the patient, I found a laceration of about three inches, with a severe contusion of the part; and a portion of the left lung protruding through the wound. I passed my finger within the opening, to ascertain whether any ribs were fractured; fortunately, they were uninjured. Having brought the edges of the wound together, secured them by four sutures, and afterwards dressed it in the usual manner, I immediately bled my patient to the extent of twelve ounces, and directed a saline cathartic to be given every hour till evacuations were procured. On seeing him in the evening, I found the medicines had induced vomiting, they were consequently discontinued, and enemas substituted every hour until stools were procured. On the following morning, September 11th, he appeared easy; no fever; his bowels had been acted upon; he was ordered to live principally upon milk and water, and that taken cold, with a little jelly occasionally. At noon, finding him restless; pulse hurried; tongue white, with difficulty of breathing; I again employed bleeding. Syncope supervening after taking eight ounces, I desisted; the enemas were repeated, and particular directions given to keep him as quiet and cool as possible. On inquiry in the evening, I found he had been much easier for three hours after the last bleeding, but latterly had become more restless, and complained of a considerable tightness across his chest, attended with difficult breathing. I again had recourse to the lancet, and took eight ounces more blood, which materially relieved him; the injections injections were repeated. On seeing him the following morning, September 12th, I found he had rested a portion of the preceding night; but, the symptoms threatening pneumonia, I bled him again to the quantity of eight ounces; enemas were repeated, and particular orders given that his diet should be continued as before. I found him, at the evening visit, much calmer than I had ever seen him before. The following morning, September 13th, he again complained of tightness across the chest. Conjecturing it might originate from the dressings, they were removed, and fresh ones applied; little or no discharge followed; he had had no rigors, yet still there was considerable heat of skin; depletion was again employed, and the same quantity taken as before, which alleviated the tightness of his breast. In the evening, he continued easier. September 14th, symptoms better in every respect; breathing more free. September 15th, no febrile symptoms present; on dressing the wound, I find it open from sloughing of its edges, and have merely dressed it with dry lint, which is retained by straps of adhesive plaster. The bark was given in doses of ʒſs. ter. in die. September 16th, no bad symptoms, but a discharge of a serous-like fluid, which passes out of the wound in considerable quantity. Contin. Cinchon. ut antea. He was ordered a more nourishing diet. September 17th,—the appearance of the wound larger from much sloughing of the pleura; his general health, however, appears good. Cont. Cinchon. cū addit. gr. x. singul. dosib. Without adverting to the minutiæ of the case, I have only to observe, that the latter treatment was pursued for a period of two months before the cavity was completely granulated. I have to remark the slow manner in which the process of granulating takes place in membranous parts; I have frequently observed it, and such was the case in this instance.

Case II.—James Robinson, labourer, ætat. 40, while employed in the coal-pits on the morning of the 18th of November last, received a severe contusion from the falling of a large portion of coal, which struck him on the superior dorsal vertebra. I saw him immediately after the accident, and found him labouring under the most distressing dyspnœa, which almost amounted to suffocation; the pulse seemed labouring, and, from his appearance, I fully believed he would not survive long, suspecting that internal hæmorrhage had taken place, although he had not expectorated much blood. From the good effects of copious depletion in the preceding case, I directly extracted thirty-two ounces of blood, and directed some cathartic bolusses to be given every hour, with a view of opening the bowels. I saw him again again at noon, and found he had breathed much better for two hours after the bleeding, but he fancied the symptoms increasing within the last hour; I again bled him to the extent of twenty-four ounces, which produced instant relief; the medicines had operated freely; he was advised to be kept cool and quiet. In the evening, the distressing symptoms had returned, which were again relieved by venesection; the quantity of ℥xvj. being taken. The following morning, Nov. 19th, he appeared easier, yet his breathing seemed hurried; pulse quick; tongue dry; skin hot. The operation was again performed, and with equal advantage in relieving his breathing. At noon, he continued easier; pulse softer, with less heat of skin. When I saw him in the evening, the symptoms continued better, and the only complaint was a troublesome cough, which had existed some time previous to the accident. For this, he was ordered the following ℞. Carb. Ammon. ℈ij. Aquæ Ammon. Acetat. Font āā ℥iiiſs. Tr. Camph. Comp. ℥i. m. Cap. ℥i. 2 da quaq. hor. I have only to mention, that the termination of this case was equally favourable with that of the preceding, which I attribute, in a great measure, to the early and free use of the lancet, a remedy which, when used in the first instance, I have generally found of singular service in these cases; it should be used freely, where it is admissible; for, if employed only partially, or locally, a kind of re-action takes place, which is succeeded by an irritable state of the system, that speedily destroys the patient, or the secondary symptoms of inflammation follow; which latter, if it does not terminate in death, at best protracts the patient's recovery to a long period.

*Barnsley; December* 11, 1816.

